# Fact vs. Affect in the Telephone Game: All Levels of Surprise Are Retold With High Accuracy, Even Independently of Facts

**DOI:** 10.3389/fpsyg.2018.02210

**Published:** 2018-11-20

**Authors:** Fritz Breithaupt, Binyan Li, Torrin M. Liddell, Eleanor B. Schille-Hudson, Sarah Whaley

**Affiliations:** ^1^Department of Germanic Studies, Indiana University Bloomington, Bloomington, IN, United States; ^2^Cognitive Science Program, Indiana University Bloomington, Bloomington, IN, United States; ^3^Department of Linguistics, Indiana University Bloomington, Bloomington, IN, United States; ^4^Department of Psychological and Brain Sciences, Indiana University Bloomington, Bloomington, IN, United States; ^5^Hutton Honors College, Indiana University Bloomington, Bloomington, IN, United States

**Keywords:** narrative, surprise, narrative affect, serial reproduction, event, cultural transmission

## Abstract

When people retell stories, what guides their retelling? Most previous research on story retelling and story comprehension has focused on information accuracy as the key measure of stability in transmission. This paper suggests that there is a second, affective, dimension that provides stability for retellings, namely the audience affect of surprise. In a large-sample study with multiple iterations of retellings, we found evidence that people are quite accurate in preserving all degrees of surprisingness in serial reproduction – even when the event that produced the surprisingness in the original story is dropped or changed. Thus, we propose that the preservation of affect is an implicit goal of retelling: merely do retellers not recall highly surprising events better, but rather they register all levels of surprisingness precisely and aim to surprise their implied audience to same degree. This study used 2,389 participants.

**Significance Statement:** Story retelling is a process whereby cultural information is transmitted horizontally across social networks and vertically down generations. For the most part, retelling research has focused on the relevance and stability of factual information, “who did what, where, when, and why”; comparatively little is known about the transmission of affective information. We suggest that affect can serve as a second axis of stability for retelling, partially independent from factual information. In serial reproduction tasks modeled after the telephone game, we find that surprisingness of stories is well preserved across retellings – even when the facts and events of the story are not. The findings are significant for the communication of information, and thereby also the stability and transformation of culture in general.

## Introduction

Cultural transmission and evolution is information-based; the study of story retelling is thus a useful tool that can reveal mechanisms by which cultural stability or innovation occurs (Varnum and Grossmann, 2017). The majority of research has focused on the relevance and stability of factual information, “who did what, where, when, and why” (Bartlett, 1932; Trabasso and van den Broek, 1985; Trabasso et al., 1989; Zwaan et al., 1995). However, relatively few studies have considered the affect of the audience in story comprehension and retelling (Eriksson and Coultas, 2014; Nabi and Green, 2015). Surprise is one such understudied affect that may be important in storytelling. It has been speculated that attention to high surprise provides an adaptive advantage (Reisenzein et al., 2012; Topolinski and Strack, 2015), and so it seems plausible that an affect such as surprise could orient story retelling and provide stability in retelling, even when other elements change. In practical terms, we hypothesize that in repeated retellings, such as in the telephone game, the “surprisingness” (how surprising a story is) of the original story would be preserved in the retold stories. In contrast to memory studies, we do not suggest that high affect (high surprise) is singled out in retelling. Rather, we suggest that all levels of affect (surprise), including low levels, are preserved and transmitted with precision. Indeed, this is what we found and we will propose a model of story retelling that adds a second axis of stability to the stability of facts (“who did what, where, when, and why?”), namely the audience affect of surprise. Moreover, we will suggest that affect can be transmitted and is often transmitted even when the factual information that produced the affect in the original story is dropped or changed.

To test our general hypothesis, we developed a task involving serial reproduction of narratives (for a discussion of methods that test cultural transmission, see Mesoudi and Whiten, 2008). We asked participants to retell short stories for us. Other studies have used binary models, which distinguish only low and high affect, or ternary models, which distinguish absence, low, and high affect. In contrast, we used a gradation of seven stories from very low to very high affect (surprisingness). These stories were almost identical, varying only in the ending, which was manipulated to be more or less surprising. Our retellers only saw one version of each short story. In our study, each story was retold in multiple telephone-game chains, each chain comprised of three different participants. We then examined the resulting versions for the presence and absence of various features, including surprisingness.

The experimental research on repeated retellings or serial reproduction of narratives was pioneered by Frederic C. Bartlett in a set of studies published in 1932 as *Remembering: An Experimental and Social Study.* Among other things, Bartlett found that retellers tend to “rationalize” the story elements that they cannot make sense of by inventing new causalities. The implication of Bartlett’s research is that highly surprising story elements would be omitted or altered to be less surprising in retellings. This theory is supported by two studies on retelling, each with different designs, definitions, and aims. Norenzayan et al. (2006) show that certain stories (those that have a few, but not too many counterintuitive events) are retold with more stability than stories with more counterintuitive events or stories with few or no counterintuitive events. Barrett and Nyhof (2001) have shown that bizarre story details suffer a high degree of entropy in retellings and often disappear, while counterintuitive story elements that violate everyday experience and expectations are more successfully transmitted [for a general discussion of minimally counterintuitive information, see Purzycki and Willard (2016) and in the context of humor, Purzycki (2010)]. Based on these studies as well as work that replicated Bartlett (critically about Bartlett, see Roediger et al., 1993; replicating and confirming, Bergman and Roediger, 1999), one might theorize that highly surprising narratives would lead to more rational story changes and that unsurprising stories would lead to more interesting distortions. By creating a seven-step gradation, we also wanted to test whether minimally counterintuitive information performs better in retelling.

Other story changes and biases in retelling have been identified that may or may not be related to surprise. For instance, Moussaïd et al. (2015) found that retellers amplified risk warnings in medical labels when they were prompted to pay attention to risk. Children of specific ages and in specific cultural contexts can amplify danger (Clark et al., 2016). Kashima (2000) observes an effect of radicalization of racial stereotypes in serial reproduction (see also Lyons and Kashima, 2006; Kashima and Yeung, 2010). Fake news (as rated by fact checkers) has been found to spread faster via social media than correct news (Vosoughi et al., 2018). The authors reason that novelty and surprise may play a role in the bias. Mesoudi et al. (2006) make a case for social-information bias in retelling, though their data might also be interpreted as more simply showing a preferential treatment of narratives in retelling versus more factual information. Since narratives concern social information, the difference may not be significant in cultural transmission. In addition, Marsh (2007) suggests that people can activate a schema when retelling a story, such as the “bad roommate schema,” thereby producing errors and omissions (Marsh, 2007: p.18). For an overview of some more transmission biases see Mesoudi and Whiten (2008).

There are, however, reasons to believe that surprisingness might behave differently from the above-listed types of narrative features (e.g., bizarreness of specific text elements, clustering of counterintuitive events, risk, etc.) since surprisingness emerges in response to audience expectations. The audience of a story forms ongoing expectations and predictions of what might happen while reading or listening (Rapp and Gerrig, 2006; Ely et al., 2015), and these expectations may or may not be met, resulting in higher or lower surprisingness. This means that “how surprising a story is” is not simply an impression based on the presence or absence of surprise markers, but rather an appraisal of the narrative as a whole. Therefore, we speculate that surprisingness of otherwise similar stories may not be as subject to change and regression to the mean as other previously-studied story features. We also reason that retellers are sensitive to how surprised a story makes them, and that retellers aim to recreate that level of surprise in the audience of their retelling.

Story retellings, story processing, comprehension, and memory are not only shaped by internal story elements, but also paratextual information. For example, whether a narrative is perceived as factual or fictional has been shown to influence memory accuracy (Zwaan, 1994) and reading time (Altmann et al., 2014), but not absorption or immersion (Hartung et al., 2017). Instructions to participants also shape retelling. For example, prompting retellers to be more accurate results in more detailed retellings as well as better memory of the retold story (Dudukovic et al., 2004). By contrast, we do not prompt or prime retellers to pay attention to facts, affect, or surprise. Audience effects can also influence retelling. It has been found that both written forms of recall (even in the absence of an immediate audience) and oral forms of recall can produce similar effects (Barrett and Nyhof, 2001). Audience effects are not to be confused with audience *affects*, such as audience tuning to only imagined audiences (for an overview see Hirst and Echterhoff, 2012).

Story retelling is different from recall. While recall is focused solely on accuracy, retelling also involves multiple audience-focused goals. These can include entertaining, conveying affect, or evoking an affect in the audience, all of which can produce deliberate inventions and distortions (Marsh, 2007). Which goal and which story elements are emphasized is largely shaped by expectations about the audience and other paratextual information, and such audience effects have been found to have implications for accuracy (Dudukovic et al., 2004; Marsh, 2007; Hirst and Echterhoff, 2012). The differences between retelling and recall mean that we do not know whether findings of recall studies apply to retelling studies. We suggest that they are not fully applicable, specifically with regard to emotions. There is broad consensus that emotions play a more prominent role in recall than other forms of information (Strongman and Russell, 1986; Posner and Snyder, 2004). Research also suggests that emotional or affective content *enhances* recall (Bower, 1981; Doerksen and Shimamura, 2001). However, what is less known is to what degree minimal affect and emotion are favored in recall or retelling. We suggest that even minimal affect is preserved in retelling – even as other information is omitted – and that affect plays a significant role in story construction.

Our studies do not give the participants a choice of which story to retell. Scholars examining urban legends distinguish between three phases of transmission process: a choose-to-receive phase; encode-and-retrieve phase; and a choose-to-transmit phase (Eriksson and Coultas, 2014). Studies have found that participants prefer to pass along stories that contain strong emotions (disgust) more than stories with weaker emotions (Heath et al., 2001; Eriksson and Coultas, 2014); likewise information that lacks social content is chosen less often for transmission (Mesoudi et al., 2006; Stubbersfield et al., 2015). False or fake news are also selected at higher rates for transmission (Vosoughi et al., 2018). By excluding the first phase of transmission, our studies compliment these studies by allowing comparisons between these phases.

Observing transmission in storytelling provides insights into the cultural dynamics that lead to the stabilizing of culture. It is therefore of great interest to understand which information, including affective information, is passed on and which information is dropped or changed. This process is far from mechanical: computational approaches of story generation, for example, face difficulties accounting for a multitude of features such as focalization, audience effects, and creativity, which distinguish human retellings from computational attempts at story generation and stabilization (Gervás, 2009). There is evidence that affective communication is understood cross-culturally (Gudykunst and Ting-Toomey, 1988; Scherer and Wallbott, 1994). There is also some evidence that story-fact recall is similar cross-culturally (Mandler et al., 1980). However, we do not know whether the nature of affect retelling is cross-culturally similar. It seems likely that a fine-grained analysis would show differences. Tsai et al. (2006), for example, reveal that there are cross-cultural differences between actual affects and ideal affects (i.e., how people want to feel) and that cultural factors have a bigger impact on the ideal affect than on the actual affect. This difference suggests that cross-cultural patterns might emerge in which affects people choose to retell and in which intensity based on how closely the affect aligns with their ideal. Our study focuses exclusively on American adults and therefore does not allow cross-cultural comparisons, but we do report differences in gender and age. However, we plan to conduct cross-cultural studies in the future.

### Surprise, Affect, and Narrative

What is peculiar about recreating audience surprise in retelling is the temporal dimension of surprise. Surprise occurs from the discrepancy between what is expected in a certain situation and what actually happens. Hence, in retelling surprise, retellers have to recreate a before-and-after effect that is reminiscent of the original story. Retelling surprise thus involves recreating a sequence of events or inventing a new sequence of events that will cause audiences to be affected in certain ways.

Since Bartlett (1932), the majority of research on story retelling and story comprehension has focused on how the factual content of narratives is stored and retrieved (for an overview, see McNamara and Magliano, 2009). Yet affect appears to be a central aspect of narration, and evolutionary psychologists have suggested that communicating emotional information is key to human beings as language users (Dunbar, 1996; Tomasello, 2009).

In the standard literature, surprise is regarded as an affect (e.g., Reisenzein et al., 2012). More specifically, surprise is an affective or emotional reaction to an unexpected or improbable event to which it is adaptive to pay attention (Reisenzein, 2012; Topolinski and Strack, 2015). The affect connected with surprise appraisal is typically described as an emotional excitement (Burgoon, 1993; Teigen and Keren, 2003).

There are competing views about the precise link between probability judgment and affect. The common view, in line with the appraisal theory of emotion, holds that emotions follow from the appraisal or judgment of the situation (see for example Reisenzein et al., 2012). It has also been suggested that in the case of surprise the affect could lead to a judgment (in line with the two-phase model by Noordewier et al., 2016).

People react with high consistency to changing surprise levels (Grimes-Maguire and Keane, 2005), though most studies do not distinguish between different degrees of surprise. Regarding surprise as a reaction to improbable occurrences, different views exist related to probability judgment or appraisal. Surprise has been explained as a violation of probability or probabilistic reasoning (Reisenzein, 2000), or as a gap in cause and effect (Kahneman and Miller, 1986), and is often associated with counterintuitive or schema-inconsistent information (Norenzayan et al., 2006). Maguire et al. (2011) make the case for a difference in cognitive processing between probabilistic and cause-and-effect reasoning. Some models measure degrees of surprise as a one-dimensional distance to expectations or distance to “last period’s belief” (Ely et al., 2015).

It is not clear what valence surprise has or whether this valence is consistent across all cases of surprise. Some argue that surprise is negative (Topolinski and Strack, 2015), some that it is neutral (e.g., Reisenzein, 2000) and others that it is positive (Fontaine et al., 2007). Noordewier et al. (2016) suggest that the different evaluations of the valence of surprise are due to temporal processes within surprise that begin as negative states (violations of expectations) and lead to more positive states in a sense-making process. Another possibility is that the degree of surprise impacts valence.

In our retelling studies, surprise is tied to narrative events (see also Norenzayan et al., 2006). Successful narrative events tend to be marked by coherence, relevance, meaningfulness (Schmid, 2010), surprise, vivacity (Rapp et al., 2011), and typically involve a before-and-after effect (Schmid, 2003; Hamilton and Breithaupt, 2013). One should note that there can be tensions between surprise and coherence. It is possible that coherence is an ex-post category that can integrate surprising events since surprise can stimulate processes of sense-making, reframing, and contextualization (Noordewier, 2016). In the context of narratives, surprise may also be linked to interestingness or suspense (Silva, 2009; Schmid, 2010; in music: Margulis, 2014), event significance, and vicariousness/transport/empathy (Keen, 2006; Green and Donahue, 2009). In general, there is significant evidence for the privileging of events in cognition (see, for example, Radvansky and Zacks, 2014).

### Research Questions and Hypotheses

The goal of the study is to examine how different degrees of surprisingness in stories impact story retelling. Specifically, we ask:

(1) How accurately is surprisingness preserved over retellings; is there an accuracy difference between stories of high-, low-, and mid-level surprisingness? We hypothesize that retellers will be sensitive to all levels of surprise, but we predict that retelling will alter surprisingness.
(2) How accurately are facts and events preserved over retellings? Do different degrees of surprisingness lead to different qualities of factual retelling? Based on previous research, we hypothesize that there will be significant omissions of factual information. We also hypothesize that facts tied to the main event will be preserved with higher accuracy and that stories with an optimal mid-level of surprisingness (Question 1) will be retold with higher overall factual accuracy.
(3) How is surprisingness preserved? Can surprisingness be preserved only if the original story elements are preserved or can surprisingness be preserved independently of the facts and events of the original story? We hypothesize that there will be a significant number of stories that will preserve the original levels of affect while not preserving the story facts that generated the affects in the original story.

To address these questions, we developed a serial reproduction task with short stories. We asked participants to retell these stories for us in the format of the telephone game – referred to in technical terms as serial reproduction. These stories were each retold three times, each time by a different reteller, and we asked other participants to rate the surprisingness of each original version and each retelling along with other data.

## Materials and Methods

### Participants

For the main study, we recruited participants on Amazon Mechanical Turk for several tasks:

1. 100 participants rated the surprisingness of the stimulus materials (the variations of three basic stories) in a pretest,
2. 635 participants retold these stories (participant retold one of each of the basic stories),
3. 500 participants rated the surprisingness of the retellings.

We then replicated our findings with shorter stimulus stories, reported below (and in Appendix D). This included the following tasks:

1. 42 participants rated the surprisingness of the stimulus materials (the variations of the three story sets) in a pretest,
2. 698 participants retold these stories (participant retold no more than one of each of the basic stories),
3. 192 participants rated the surprisingness of the retellings,
4. 222 participants rated the stimulus materials for additional criteria (see Appendix C).

Rand (2012) found that as many as 97% of Mechanical Turk users are trustworthy in their reporting. We included a control question (“which of the following animals says “moo”?”) and excluded participants who did not pass from taking the experiment. We should note that our study excluded juveniles below 18 and that we had few retellers above 65. Our participants resided in the United States (Mechanical Turk does not provide filters to limit access to native speakers). We also set filters so each participant could only participate once in the entire set of tasks, and we collected IP addresses to control for this. The average age of participants who performed retellings was 33.6 years old (*SD* 11), and 57% reported as female. Among the 500 participants recruited to rate the surprisingness of first-, second-, and third-iteration retellings, the average age was 34.6 (*SD* 10.7), and 51% reported as female. We paid all participants at an approximate rate of $6/hour. Participants gave informed and written consent to participate in the study and to allow us to use the collected data.

### Stimulus Materials

We generated three short stories of 13–16 sentences. Each story featured a challenging situation for the main character. For example, a student is taking a difficult test and needs help or a daughter is having an argument with her mother. For each story, we created seven variations that provided different solutions to the challenging situations, revealed at the end of the stories; these varying solutions were one or two sentences long. These variations were more or less surprising (and we asked participants to measure their specific surprise levels, as we will report). *In toto*, we used three story sets of seven variations each. We will refer to these *story sets* according to the names of their main characters, *Jason, Sarah*, and *Robert*, and we will refer to within-set variations as Jason A-G, Sarah A-G, and Robert A-G. All variations are given in Appendix A. We will use the term “basic event” for the one-to-two-sentence-long ending solution that is manipulated between the different variations accounting for their differences in surprisingness. We use the term event according to “event II” in the terms of Hühn (2009).

In the pretest, 100 participants on Amazon Mechanical Turk rated the surprisingness of the variations of the three story sets. Each participant reviewed one variation from each basic story set (Jason, Sarah, and Robert) in fully randomized order; and each original story was evaluated by an average of 14.3 participants. The instructions read: “Please tell us how surprised you are by the story below.” This was followed on the same screen by the text of the story. Below the text was a slider that participants could drag on a scale from 0 to 7 to the tenths place, allowing for answers such as 5.1. We marked 7 as “most surprised” and stated: “If you are not surprised at all, please mark it as 0.” The median surprise values are reported in Appendix B. The median surprisingness of the original story variations ranged from 1.0 to 6.1. The seven variations of each story set provided a robust and consistent ranking from low to high levels of surprise (see Appendix E for details on the differences between story variations as revealed by a Bayesian ANOVA).

To better clarify how participants conceptualized surprise, we correlated surprise with other criteria, namely probability, eventfulness, gap in cause and effect, event strength, interest/suspense, emotional excitement, or vicariousness that are sometimes used in psychological or narratological literature. Surprise was most strongly correlated with probability (-0.97) and eventfulness (0.84). Surprise levels were negatively correlated with vicariousness (-0.76). For a description of procedures and all values, see Appendix C.

### Procedures of Retelling Narratives

The findings of our pretest suggested that people are sensitive to varying intensities of surprise (surprisingness). However, we did not know to what degree people faithfully reconstruct events and surprise when retelling stories, especially when they are not prompted or primed to do so.

To study our three main questions, we used our three sets of basic stories, as presented above, 21 stories in all. For retelling, each participant received one variation, at random, from each of the three basic story sets; we also randomized the sequence in which the story sets were presented. That is, participants each retold one Jason, one Sarah, and one Robert story.

**FIGURE 1:**
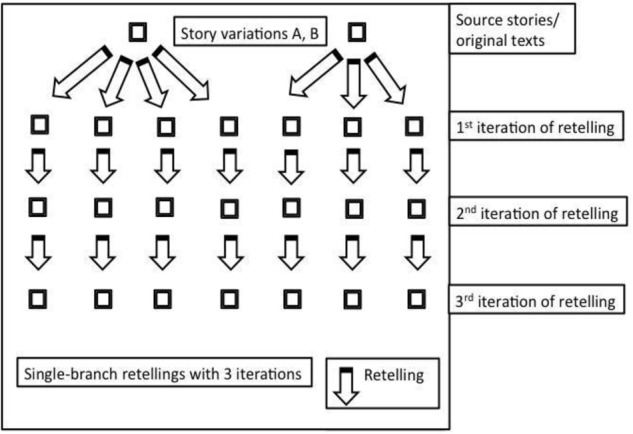
Procedures for retelling. The original texts were first retold by many participants. After the first iteration, each retelling was routed to a new reteller for the second iteration, and again for the third.

Each of the 21 variations of the basic stories were passed along telephone-game chains of three people each. A story variation, Sarah A for example, was read and retold by a participant on Mechanical Turk; the resulting story would be passed on to another participant for a second round of retelling; then this resulting story would be passed on to another participant for a third and final round of retelling. This final retelling is the product of a retelling chain of three participants, and multiple chains of retelling were obtained for each story variation. (Each participant could only contribute to one retelling chain per story set, i.e., each participant only retold one Jason, one Sarah, and one Robert story.) See Figure 1. In the end, we compared and evaluated all the different retellings that started from the same original variation in terms of surprisingness and other measures. We ended after three iterations of retelling since results stabilized markedly after the first iterations (in terms of length, reduction of the narratives to single event stories, key words, and surprise, as we will report). Barrett and Nyhof (2001) also used the standard of three iterations of retelling; Eriksson and Coultas (2014) used four iterations in their study of information deterioration. By retellings or individual retellings, we refer to the story retellings produced by individual participants.

Using a variation of Kashima (2000), we used the following general instructions for retelling: “This is an experiment about how people communicate a story to another person. We will show you the text a student wrote and give you time to read it. Your task is to remember it so that you can tell the story to another person in your own words. The next person will then communicate it to another person. It is important that you understand the text. We will ask you some questions about it later on. Please begin.” We did not prompt participants to pay attention to surprise, and it may be that participants saw the study as a memory task with accuracy as its goal. This is in line with our aim of testing the role of affect in story retelling when it is not primed. Participants who participated in retelling were asked to retell three short stories.

Participants retold the stories one by one; each time, they saw these instructions on their screen: “Please spend at least 40 s reading the following story. You will be asked to retell the story on the following page,” followed by the story, though for the shorter second- and third-iteration retellings, the directions specified 20 s. We did not specify an explicit goal for the retelling. To prevent participants who did not read the story from advancing, we set a timer so one could advance only after 40 s for the first-iteration retelling and 20 s for the shorter second- and third-iteration retellings; participants had up to 1 h for all the tasks combined. After reading the instructions and the story, participants clicked a button to proceed to a new screen where they could type in their retelling of the story they had just read.

Each story variation was retold across three iterations according to the procedures outlined above in Figure 1, which resulted in 285 first-, 285 second-, and 285 final-product, third-iteration retellings. There was an average of 14 third-iteration retellings for each story variation. For example, our study produced 16 third-iteration Jason C stories, 13 Jason D stories, etc. We then routed the resulting 285 third-iteration retellings to 500 separate participants on Mechanical Turk to evaluate the surprisingness of these stories with instructions identical to those used in the pretest. Each participant received 15 of the retellings, fully randomized, and rated surprisingness on a scale from 0 to 7. On average, each individual retelling was rated by 7.8 different raters for the first iteration, 7.9 for the second iteration, and 10.2 for the third iteration on Mechanical Turk.

We compared the surprisingness scores for all retellings by means of a Bayesian ANOVA. We also compared the median surprisingness ratings of the original stories with the median ratings of the retellings by means of Pearson’s correlations. For the ratings of the retellings, we determined the median rating of each individual first-, second-, and third-iteration retelling and then calculated the average value of all first-, second-, third-iteration retellings within the same story variations. E.g., there are 15 third-iteration retellings of Jason B. Suppose an average of 10 participants rated each of the 15 retellings. We would determine the median ratings of each of the 15 retellings, then we would take the average of these 15 ratings for the third-iteration average of Jason B.

In order to gather data on the transmission of factual content, we coded the original stories for details; 20–24 for each story variation (for studies that have used similar detail codings, see Davis et al., 2015; Moussaïd et al., 2015). After the retellings, experts counted the number of coded details present in all third-iteration retellings including for example, basic actions such as “Sarah apologizes,” verbal constructions such as “studied all night,” or descriptions such as “stern but knowledgeable professor”, also accepting non-verbatim detail representation such as “says sorry,” “worked hard,” or “strict professor.”

We wanted to know whether the varying basic events would be preserved after the retellings. (The varying basic events are given in Appendix A). Experts rated full presence, partial presence, or full absence of basic events in the third iteration, according to the following criteria:

(1) Full event presence: event resolves overall problem of the story set – lack of girlfriend/date, emotional inability to face mother, or not knowing answers to exam – by means of the key action of the story variation (wearing superhero costume, swimming in a pond with clothes on, banging one’s head exercising in the bathroom, etc.). The action does not need to be stated verbatim.
(2) Partial presence. Either: (a) the overall problem is not present, or not resolved as a causal result of the key action; or (b) the overall problem is resolved, but the key action is changed, but partially preserved (dressing up without mention of superhero costume; swimming in pond without mention of wearing clothes; walking into a wall without mention of the bathroom or exercise).
(3) Absence: no details of the key action are present.
Two experts independently coded all the stories and agreed at a rate of 98%; we excluded cases of disagreement leaving us with 280 third-iteration retellings with expert agreement.

## Results

**FIGURE 2:**
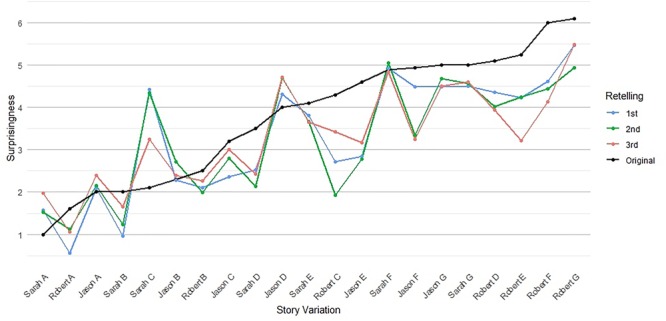
Median surprisingness of all story variations, across all iterations of retelling (Original, 1st, 2nd, and 3rd). The *x*-axis lists the code names of story variations. The *y*-axis represents the average surprise of all retellings within each variation on a scale from 0 to 7.

The surprisingness of retellings showed remarkable stability across all iterations. We calculated correlations and performed a Bayesian ANOVA. The results revealed a notably high correlation between the surprisingness ratings of the original stories and the surprisingness ratings of the stories after three iterations of retelling. [Jason stories: Pearson’s *r*(97) = 0.69; Sarah stories: Pearson’s *r*(94) = 0.86; Robert stories: Pearson’s *r*(94) = 0.93]. Each story variation was evaluated on average by 10.2 participants. For all results, see Appendix B. Figure 2 shows the overall surprisingness correlations between texts across all iterations. The surprisingness correlations of adjacent iterations of retelling – i.e., between first–second and second–third – were higher than the correlations between non-adjacent iterations – i.e., between original-second and first–third, with one exception in the Robert story set (see Appendix B).

The standard deviation of the difference between original-first iteration median surprisingness ranged from *SD* = 1.26 to *SD* = 1.47 (Sarah and Robert story sets, respectively). The standard deviation of differences between first–second iteration and between second–third iteration median surprisingness fell in a similar range: *SD* = 1.1 to *SD* = 1.5 on the 0–7 scale. On initial inspection of the median surprisingness values, it appeared that the highly surprising original stories were retold as slightly less surprising retellings, but this pattern was not detectable statistically; we directly address the changes in surprisingness ratings across iterations below.

### Question 1: On the Preservation of Surprisingness

In order to validate the apparent pattern of surprise maintenance, we performed a Bayesian ANOVA on the surprisingness ratings of all individual retellings (not the median surprisingness ratings that have been presented in the previous descriptive statistics and in Figure 2). This approach is similar to a traditional ANOVA, with a two-factor 21 (story) by 4 (original-third iteration) design. However, the Bayesian approach has multiple advantages. The hypothesis in question, surprisingness maintenance, is essentially a null effect, in that it predicts little to no change in surprisingness ratings from the original story ratings to the third-iteration ratings. Traditional frequentist ANOVA can never truly provide support for such a hypothesis, it can only fail to reject this hypothesis. Moreover, the Bayesian approach allows us to avoid any concern with unbalanced sample size across conditions. Finally, assessing the surprisingness maintenance hypothesis involves several planned comparisons across levels; in traditional frequentist ANOVA this would require *p*-value adjustment for multiple comparisons. In contrast, a Bayesian approach does not use constant Type I error rate as its method of evaluation, it provides the best posterior estimate given the data and this posterior distribution does not change based on the comparisons made by the researcher. For a complete description of Bayesian ANOVA see Ch. 20 in Kruschke (2015), and for a more general comparison of Bayesian and frequentist approaches, see Kruschke and Liddell (2018).

Bayesian ANOVA does not utilize an omnibus test of main effects, and as such we move directly to the comparisons of interest: changes in surprisingness ratings across the retelling factor. To assess this, we compared the posterior estimate effects of original, first-, second-, and third-iteration retelling on surprisingness ratings. For each comparison we computed a 95% highest density interval (HDI) on the difference between the two iteration conditions, which is analogous to a confidence interval in that it is a range of reasonable values, but is computed from the Bayesian posterior distribution. All comparisons are presented in this manner on a standardized effect size (analogous to Cohen’s *d*) scale, and we used a region of practical equivalence (ROPE) around 0 of -0.1 to 0.1, as this constitutes half of the typical cutoff for a “small” effect size of 0.2 (for more on effect size ROPEs and HDIs, see Ch. 12 of Kruschke, 2015). We compared the 95% HDIs to this ROPE to determine if the difference is different from zero (HDI entirely outside the ROPE), equivalent to zero (HDI entirely within the ROPE), or inconclusive (HDI crosses the ROPE limits).

Differences between first–second iteration retellings (95% HDI -0.016 to 0.096), first–third iteration retellings (95% HDI -0.061 to 0.046), and second–third iteration retellings (95% HDI -0.088 to 0.026) are all equivalent to zero. In other words, we have evidence that surprise did not change from the first iteration to the third. When comparing the original surprisingness ratings to first- (95% HDI 0.052–0.309) and third-iteration ratings (95% HDI 0.052–0.309) the results are inconclusive, but there is a consistent trend of a small decrease in surprisingness (modal estimate of 0.18 on the effect-size scale, or 0.35 on the original 7-point scale) across all iterations, when compared to the original stories. But as noted, this decrease is so small that the HDI crosses the ROPE limits and thus the trend is not clearly detectable. Thus, the general conclusion regarding maintenance of surprisingness is that there is perhaps some weak evidence for a decrease in surprise *in the initial iteration of retelling only* and after the first iteration, surprisingness is remarkably consistent across further retellings.

The ANOVA findings concerning the significance of the first retelling and the potential insignificance of further retellings with regard to surprisingness are, in large part, corroborated by the correlation values. The correlations of median surprisingness ratings from original-to-first-iteration retellings range from 0.73 to 0.95 across story sets, whereas the correlation of the other retellings from first-to-second iteration and second-to-third iteration range from 0.84 to 0.98. The correlation of surprisingness ratings using all stories (not medians) were 0.62 for the first retelling, 0.71 for the second retelling and 0.75 for the third retelling (see Appendix B). These data give some evidence that story retelling stabilized after the first retelling.

Put differently, after the first retelling, changes in surprisingness were small. (There was a weak trend of further stabilization from the second to third retelling for the Jason and Sarah stories, whereas the Robert stories had a lower correlation of surprisingness values between the second and third retelling than between the first and second retellings). The average values for each story variation are given, in Appendix B.

### Question 2: On the Preservation of Facts and Events

In terms of information accuracy, the resulting third-iteration retellings from the stories had an average length of 277 characters overall, a shrinkage of more than 77% from the original stories. The average detail preservation rate among all third-iteration retellings was 23.5% or about 5–6 details out of the original 20–24. We also charted retellings that dropped the original basic event entirely. Experts agreed at a rate of 98% when discerning these stories and we excluded cases of disagreement. Of the 280 third-iteration retellings with expert agreement, 50.3% maintained the key elements of the basic event, 18.9% maintained partial elements of the event and 30.7% lost all aspects of the basic event.

The slight length difference between the original stories was not a significant factor for lengths of third-iteration retellings. The correlations between the lengths of original stories (e.g., Robert A) and their third-iteration retellings (e.g., retellings of Robert A) were low across story sets (0.11–0.22), and we concluded that length of the original stories was not a significant factor for resulting third-iteration length. Thus we ruled out the slight length differences between the original stories as a confounding variable for the length of third-iteration retellings. Also, typically, the first-iteration retelling was decisive in shrinking the overall story length and in detail reduction, average length shrinkage was 55.2% in the first iteration.

### Question 3: On the Preservation of Surprisingness Independent of Facts and Events

Perhaps our most interesting results concern stories that had dropped the original basic event entirely. Astonishingly, even stories that did not maintain the basic event preserved the surprisingness of the original story to some degree. Since the variance in surprisingness is linked to the varying basic events, one should expect the retellings without event presence to not correlate at all with the original surprisingness ratings. Visible in Figure 2, the story variations of all retellings have marked differences with regard to surprisingness and they form a gradated slope of low to high surprisingness. One should expect the retellings without the event to not establish a similar slope from lower to higher surprise levels analogous to the original stories, but to be flat instead. However, the surprisingness of the retellings in which the basic event had fully disappeared still record notable correlations between original stories and individual third retellings for two of the story sets [Jason *r*(35) = 0.46, Sarah *r*(24) = 0.15, Robert *r*(27) = 0.43; overall Fisher *z* = 0.37], see Table 1. (As noted, the Sarah set has the smallest *n* for the *event-absence* condition with only 24 stories). Given that these stories did not include the event, we should have expected a number close to 0. Still, the extremes are less pronounced for stories without event presence than for stories with event presence (for the stories without event presence, on a 7-point scale, the range was just 0.55 for the Jason stories, 1 for Sarah, and close to 2 for Robert, while the range was between 3 and 5 for the stories with full event presence). Basic events that were more surprising had a slightly higher chance of being maintained: The correlation between surprisingness ratings and event maintenance was 0.47 (third-iteration retellings), see Table 2 below.

**Table 1:** Pearson correlations between median surprisingness ratings of the original stories and median ratings of third-iteration retellings sorted by event presence.

	Full event presence (*n*)	Partial event presence (*n*)	Event absence (*n*)	Correlation full-absence (*n*)	Fisher *z* (full-absence)	*p*-value (full-absence)	Correlation full-partial (*n*)	Fisher *z* (full-partial)	*p*-value (full-partial)	Correlation partial- absence (*n*)	Fisher *z* (partial-absence)	*p*-value (partial-absence)
Jason	0.74 (48)	0.43 (9)	0.46 (35)	0.5 (21)	1.68	0.094	0.62 (9)	1.2	0.23	0.97 (5)	-0.19	0.84
Sarah	0.83 (46)	0.58 (24)	0.15 (24)	0.14 (17)	2.86	0.004^∗∗^	0.8 (17)	2.45	0.015^∗^	0.54 (13)	1.63	0.102^∗^
Robert	0.86 (47)	0.82 (20)	0.43 (27)	0.38 (25)	3.08	0.002^∗∗^	0.89 (13)	0.54	0.59	0.32 (10)	1.41	0.158

*^∗^P < 0.05 or better, ^∗∗^P < 0.01 or better.*

**Table 2:** Pearson correlation matrix of surprise and other story measures.

	Surprise	Event	Details	Length
Surprise	1	–	–	–
Event	0.47	1	–	–
Details	0.09	0.2	1	–
Length	0.24	0.29	0.71	1

Using a tool provided by Lee and Preacher (2013), we tested the significance of event preservation. The correlations between the third-iteration surprisingness ratings and the original surprisingness ratings were compared based on the level of event preservation – i.e., retellings that did not preserve the event were grouped together, likewise for retellings that partially preserved the event, and retellings that fully preserved the event. As these correlations utilize the same original surprisingness ratings the correlations are dependent, and so we tested for the difference between dependent correlations to make the comparisons. We adjusted for unbalanced sample sizes. Table 1 shows the correlations and the Fisher’s *z*-transformation for correlation coefficients. For two of the three story sets, the correlation coefficient of surprisingness between the retellings with full event preservation and event absence are significantly different. The original to third surprisingness correlation for retellings with partial event presence is in most cases not significantly different from the stories with full event presence or with event absence (in the case of the Jason set, the *n* is too small to make confident statements).

There is further evidence that some surprise was maintained without the full event; we can see this in the partially preserved condition. These stories also show significant correlations between original and third-iteration surprisingness [Jason *r*(9) = 0.43, Sarah *r*(24) = 0.58, Robert *r*(20) = 0.82].

**FIGURE 3:**
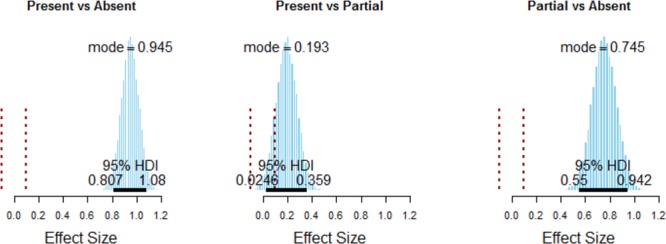
Posterior estimates of the effect size of the differences between event presence conditions. These plots show the size of the differences in surprisingness across the three event maintenance types, relative to the variability of the surprisingness ratings (i.e., on the same scale as Cohen’s *d*). The 95% HDIs are indicated by the black bar with labeled end points. For comparison, we also show the ROPE around 0 effect from –0.1 to 0.1 on the effect size scale in dotted red. The Present versus Absent and Partial versus Absent comparisons are large and far from zero, whereas the Present versus Partial comparison is trending positive but crosses the HDI limits and so is inconclusive.

We also assessed this question in a Bayesian ANOVA framework in two different ways. First, we assessed the effect of event maintenance on overall surprisingness. We performed a two-factor 21 (story) by 3 (event presence; full, partial or absent) Bayesian ANOVA to address this question. Note that this ANOVA does not include retelling iteration as a factor, as we only assessed event presence in the third-iteration retellings. The primary effect of interest was the effect of event presence. Figure 3 shows the comparison of the three conditions in detail. The overall conclusion is that stories where the event was completely absent are quite a bit less surprising than the stories where the event was partially or completely present.

**FIGURE 4:**
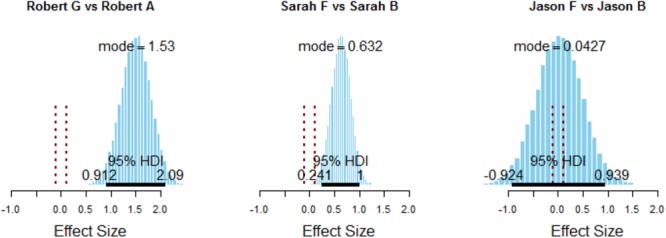
Posterior estimates of effect size for comparisons of the most and least surprising initial variations for each of the three story sets. These plots show the size of the differences in surprisingness across the most and least *initially* (before any retellings) surprising variations, at the third-iteration retelling, *only including retellings that completely lack the primary event.* As in previous plots, this difference is displayed relative to the variability of the surprisingness ratings (i.e., on the same scale as Cohen’s *d*). The 95% HDIs are indicated by the black bar with labeled end points. For comparison, we also show the ROPE around 0 effect from –0.1 to 0.1 on the effect size scale in dotted red. Note that for the Robert and Sarah stories there is a large, credibly non-zero difference in surprisingness rating, whereas the difference in the Jason stories is centered at zero, but also highly uncertain with a wide HDI.

**FIGURE 5:**
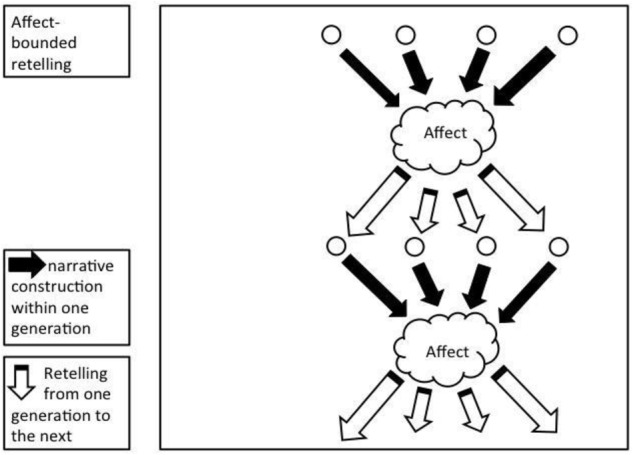
Affect-bounded retelling. In affect-bounded iteration, the story elements together cause an affect. Retellers aim to reproduce this affect and can introduce new story elements toward this end.

We also wished to assess whether there was *any* maintenance in surprise for stories that completely lacked the event, analogous to the correlation analysis for stories categorized by event absence as presented above. If so, this suggests that there is some affective information being maintained despite a complete lack of the event initially attached to this affect. To answer this question in the Bayesian ANOVA framework, we performed the full two-factor 21 (story) by 4 (original to third retelling) ANOVA presented earlier, but only on the subset of story chains that lose the event entirely by the third retelling. We then investigated whether there were still clear and credibly non-zero differences across more and less surprising stories in the third retelling, as predicted by the ANOVA model. Specifically, we compared the most originally surprising story variation to the least originally surprising story variation (i.e., comparing the rightmost and leftmost variation from Figure 3 for each story set). Figure 4 shows these comparisons in detail. This analysis demonstrates the following: the Sarah and Robert comparisons show a large difference in surprisingness rating favoring the story that was initially most surprising, whereas the Jason story is inconclusive but does not show evidence of this surprisingness maintenance. So we have strong evidence that there is surprise information being maintained independent of event maintenance, but this is not the case for all stories (see Figure 5).

There was only a weak correlation between event presence/absence and specific degree of surprisingness. There was a slight tendency to fully preserve basic events with higher surprisingness [Pearson *r*(280) = 0.47, *p* < 2.2e-16]. There was almost no correlation between detail preservation and surprisingness levels [Pearson *r*(281) = 0.09, *p* < 0.149]. Character count had a slightly higher correlation to surprisingness [Pearson *r*(281) = 0.24, *p* < 0.00006312].

### Replication With Shorter Stories

We also ran a comparison of shorter texts, based on the same story sets. These texts were much shorter and more centered on the final event variations. We followed the general procedures of the study, but only measured surprisingness for the original short texts and the third-iteration retellings. We used a total of 698 retellers and 192 raters. Again, we found a very high degree of surprise preservation at all levels, going hand in hand with event preservation. Event preservation was near 100%. Story length was reduced by around 50% over three iterations. All stimulus materials and results are given in Appendix D. This post-study with shorter text again confirms the salience of events and surprisingness levels.

### Demographics

Demographic differences did not turn out to be decisive for the surprisingness ratings or the quality of the retellings, but revealed some variations. We collected data on age, gender, reading habits, education, but not race. Overall, the individual stories retold by female retellers were 3.6% longer than those retold by males (in the first retelling, the difference was an average length of 562.56 characters for females and 542.93 for males). The cumulative effect over three iterations created a 16.7% difference in length (286.2 average number of characters for three iterations by only female participants versus 245.3 characters for three iterations by only males). Interestingly, the gender specific length reductions did not impact the surprisingness ratings which overall were close to identical for both sexes. Stories retold exclusively by women showed some increase in average details per retelling when compared to stories retold exclusively by men (across story sets this increase ranged from 25 to 38% after three iterations).

Age did not turn out to be a significant factor in measures of length or surprise preservation. We should note that our study excluded juveniles below 18 and that we had few retellers above 65. Within the bracket of 18–60 year olds, there was essentially no correlation between age of retellers and length of retellings. However, across three iterations, the varying effects of age are detectable, though not pronounced. Retelling chains primarily featuring older retellers (average age of 45 or older over three iterations), yielded third-iteration lengths which were 9.7% shorter than the average third-iteration retelling. Still, these retellings showed few other signs of deterioration in terms of event preservation, surprise preservation, or preservation of the 20–24 details we coded. Davis et al. (2015) have shown in a study on gist and detail that older populations (above 60) focus on gist. This could explain why the slightly shorter stories by older retellers in our studies preserved surprise affects at the same rate as younger retellers, since surprise is tied to the basic event and thereby likely to be captured as gist (for age differences in retellers, see also Barber and Mather, 2014; for narrative complexity in children, see Kulkofsky et al., 2008).

Individual differences in working memory capacity have been found to influence recall and textual comprehension (Daneman and Carpenter, 1980; Just and Carpenter, 1992). While we do not explicitly control for memory, working-memory-capacity differences are normally distributed (Conway et al., 2005), and our data suggest homogeneity of memory ability in our participants. Indeed, the standard deviation for preserved story facts (as indicated, we coded 20–24 details per story) ranges from *SD* = 2.02 to *SD* = 2.35 after three retellings for the three story sets. With 2,389 participants in our complete set of retellings, it is unlikely that our results are skewed by outliers.

## Discussion

Events and surprise were conserved to a remarkable degree considering that retellers were not prompted in any way to focus on these story dimensions. A highly interesting finding is that even the retellings that dropped the original surprising event maintained the surprisingness of that very event to some degree. While stories were shortened by 77% on average, story surprisingness remained highly stable between the original, first-, second-, and third-iteration retellings; this was true regardless of higher or lower original surprisingness. The majority of retellings preserved the basic event either fully or partially; but roughly only 25% of stories’ original factual details were present in the third-iteration retelling: on average, 5–6 remained out of 20–24 original details. Typically, the first-iteration retelling was most significant for changes in length, detail, and surprisingness. There was no evidence for significant change in surprisingness in the second- and third-iteration retellings, even though these iterations continued to shrink stories and reduce the number of details.

In response to our Question 1: “how accurately is surprisingness preserved over retellings; is there an accuracy difference between stories of high-, low-, and mid-level surprisingness?” We hypothesized that surprisingness would converge to an optimal mid-level over the course of retellings. However, we found that original, first-, second-, and third-iteration retellings correlated highly in terms of surprisingness and levels of surprise were steadily maintained. This effect was evident for all levels of surprisingness. A Bayesian ANOVA provided further evidence that surprisingness does not change significantly with retelling. This effect was identifiable across the range of surprisingness: we could not identify different patterns of behavior for highly, modestly, or minimally surprising stories; there was also no general trend toward high-, low-, or mid-level surprisingness. In contrast to previous studies with different study designs and definitions (Barrett and Nyhof, 2001; Norenzayan et al., 2006), *all* levels of surprisingness were preserved to a high degree between the originals and the third retellings in our study. (Although we did not explicitly test minimally counter-intuitiveness as mid-level surprisingness, our results do not provide evidence for better performance of any minimally counterintuitive surprisingness level in story retelling).

In response to our Question 2: “how accurately are facts and events preserved over retellings?” We hypothesized that factual information would get lost in the retellings, but that facts closely tied to the main event would be preserved better. We also hypothesized that stories with an optimal, mid-level of surprisingness would be preserved better overall. Much factual information was indeed lost; on average, only 25% of original story details were preserved in the third-iteration stories. Moreover, the third-iteration stories also only preserved around 23% of the original text length, but nonetheless the majority of third-iteration stories preserved the original basic event – either fully (50.3%) or partially (18.9%), giving some support that retelling is structured around events. As noted above, there was no such “optimal” level of surprisingness. There was almost no correlation (*r* = 0.09) between detail preservation and surprisingness levels.

In response to our Question 3: “can surprisingness be preserved … independently of the facts and events of the original story?” Our hypothesis was confirmed; we found evidence for surprise maintenance independent of the basic event. The study indicated that surprisingness levels are modestly preserved (Fisher *z* = 0.37) even when the event, the basis of the original surprisingness ratings, dropped out of the retold stories completely. Furthermore, a Bayesian ANOVA compared the surprisingness ratings of particular event-absent third-iteration retellings – retellings which were originally the most and least surprising variations within the three story sets. The analysis gave strong evidence that the original difference in surprise rating was maintained for two of the three story sets. These findings provide evidence that surprisingness levels can be salient and preserved independently from the facts of the basic event. Further evidence supporting these findings comes from the third-iteration retellings that only partially kept the original event (18.9% of all third retellings), while correlating strongly to the original surprisingness levels. These partially preserved-event stories contributed strongly to the overall increasing slope of surprisingness among the third-iteration retellings.

Here is an example of a retelling that morphed the original event – finding a love letter from the teacher with the answers to an exam (Robert G), rated as 6.1 on a 7-point scale of surprisingness – into another more plausible, but still highly surprising event: “Robert had two tests on the same day, a history test and a physics test. He forgot about the history test and, after he passed his Physics test, he panicked and decided to steal the answer sheet for the history exam, and he cheated his way to an A.” [rated as 5.9]. This case of the transformation of the event generally follows Bartlett’s rationalization effect in reproduction (Bartlett, 1932), while at the same time adding highly surprising elements that led to surprisingness stability across the retellings. The original event (receiving a love letter) is translated into another event that seems more plausible (“steal…cheat”), while maintaining high surprisingness in the event, and by exaggerating the affective dimension elsewhere in the story (“panicked”).

We reason that there are two possible ways in which affect levels can be preserved despite the mutation or disappearance of original story facts and events. The retelling can shift the story’s locus of surprise to a different event or element in the story, or it can morph the original event radically, replacing the details of the original event with other details. Both modes of retelling are discernable in the quoted example. The addition of Robert’s “panicked” action shifts the surprise to an earlier event in the story, while *stealing the answers* seems to have mutated from *finding a love letter*. In both cases, the probabilistic aspect of surprise is tied up strongly with the original context, i.e., an unlikely event; taken together, one can envision the affective dimension of surprise providing stability for the retelling.

How does this effect of maintaining surprisingness while dropping the specific event come about? Here is a potential explanation. Even one surprising event is enough to cause participants to see the overall story as surprising. It is this overall sense of story surprisingness that people seek to recreate, leading them to reproduce a surprising story, even as the facts and events of the original story shift. Likewise, an unsurprising story gets retold as an unsurprising story, even if the facts are not accurately represented. In this case, the reproduction of surprise acts as an implicit, though not necessarily conscious goal for retellers, namely *to surprise* their audience to the same degree that the original story surprised them.

We hypothesize that in the absence of explicit goals and an immediate audience, the perpetuation of surprise – and perhaps similar affects – guides story retelling as an implicit goal (see Figure 5). The goal of retelling though, broadly defined as “internal representations of desired states” (Austin and Vancouver, 1996), can be set by direct instructions. A reteller can be prompted for accuracy or can be motivated to “entertain” his or her audience, and each leads to different results (Dudukovic et al., 2004). Our study’s instructions did not specify a purpose for the retelling nor did it prime for surprise. We only instructed participants to remember the story well enough to retell it.

In this sense, the goal of retelling a scary story is to scare the audience; a funny story’s goal is to make the audience laugh; and a surprising story’s goal is to surprise. We describe the role of surprise as *goal* rather than *schema* because of the former’s dependence on an implied audience. Also, two rather different stories might be able to achieve the same goal of surprising the audience, even though they cannot be described as similar and falling under the same schema. The difference, however, may not be significant from the viewpoint of the reteller who might use a schema of a scary story or a mildly surprising story for retelling (see Marsh, 2007).

Overall, we suggest that story retelling employs these two core strategies to construct narratives: stories can be constructed around a narrative event and they can be constructed around a core narrative affect, like surprise. In most cases, the two go hand in hand. The narrative event does not only consist of a set of facts – who did what to whom how when and why – but also causes affective reactions in recipients; and perhaps the extent to which narrative events are remarkable at all is because they are, in part, the seat of narrative affects. This idea is consistent with the data, which show that affect preservation is highly correlated with event preservation. However, our study presents evidence that these two core strategies can also be divorced.

As highlighted, we recorded a significant portion of cases (30.7%) in which the event disappeared entirely or was radically transformed by the stage of the third-iteration retelling while the overall surprise level was modestly preserved. For this reason, we propose referring to this phenomenon as surprise- or affect-sensitive or affect-bounded retelling, i.e., using affect as a basis of story construction.

Thus, we suggest that story retelling uses both the facts of the event and the story’s surprisingness as the organizing measure guiding the retelling. If retellers understood the task to retell stories in their “own words” as a memory task with accuracy as an implied goal, this implied accuracy did not exclude surprise. We have suggested above that surprise may serve as an (additional) implicit goal for retelling when no explicit goal is provided. According to this interpretation, narratives serve as capsules to preserve and transmit surprise and perhaps similar affects.

An alternative, though related, explanation of why surprise is preserved would be that the affect of surprise “colors” the entire story in such a way that the affective mood in the end is what gets retained in recall and retelling. Memory studies involving the emotional or affective dimensions of words have shown that emotional or affective valences of words can be better remembered than the actual words (Schacter and Worling, 1985; Koriat et al., 2000). However, retelling narratives that produce narrative affects is different from remembering words. In order to accurately recreate surprise, a similar level of probability or improbability has to be created; retellers need to (re)create a context that signals the probabilities of certain events along with the surprising event.

Hence, overall, we argue that retelling is event-focused, surprise-sensitive or surprise-bounded and, in the absence of explicit goals, uses narrative affect, as a goal. Whereas the preservation of events and the affect typically go hand in hand, we collected evidence that many retellers reproduced surprisingness with some accuracy despite omitting or changing the relevant facts. These findings suggest that some factual errors in retelling are not accidents or failures on the part of the reteller, but rather the side effect of the reteller’s effort to reproduce affect accurately. Story retelling thus may be a two-pronged affair between facts and affects.

One last interpretation of our findings is through the lenses of data compression in memory. Typically, information does not need to be transmitted with full accuracy: we see this in the reduction of a story to its gist, a paper to its abstract, a high-resolution image to a thumbnail (see Koriat et al., 2000). Similarly, our results suggest that our minds compress information in the context of story retelling as original story lengths are shrunk down and details that are perceived to be irrelevant disappear. The highest resolution image of a story includes all the words, details, events, and affects. And from our data, one can argue that events and affects are the thumbnails that are transmitted as periphery words and details are filtered out. From this perspective, our finding that some stories preserved surprisingness while changing the affect-inducing events suggests that surprisingness can be a more fundamental level of resolution for some retellers than events – where surprisingness may function as a basic signal of original vs. unoriginal information; however, this final point is speculative.

This study also shows that the first retelling is of most significance for the preservation of surprise levels. Similarly, Nahari et al. (2015) found that the first reteller of a serial reproduction chain of narratives had the highest impact on the following iterations. As reported, there was a drop in surprisingness of 0.35 on a 7-point scale between the original story and the first-iteration retelling for all levels of surprisingness, low, mid, and high. For the second and third iterations of retelling there was no evidence of significant change. It is not clear why the first iteration has so much significance, but we reason that it brings the story into a form that is better suited for similar reproduction in further retellings. It is not clear whether the slight drop in surprisingness between the original and first retelling is significant or how it can be explained. Perhaps the language of the retellers is closer to the language preferred by future retellers. Or perhaps the smaller number of details lowers levels of surprisingness.

Our studies have some clear limitations. This study alone cannot comment on the precise relationship between fact preservation and affect preservation. It is not clear which side dominates or whether some people focus on facts, while others focus on affects.

In addition, we focused on short narratives and have not yet tested for other narrative structures. Our stories did not include perspective shifts (even though some retellings did). Our original stories did not explicitly state the emotional states of the protagonists. Hence, we cannot discuss whether narratives with explicit emotional words are retold differently from others [as suggested by Kensinger and Corkin (2003), in the case of short phrases]. We did not distinguish between long-term recall and retelling, but instead focused on immediate retelling only.

We have suggested that the affective side of surprise plays a role in the effect of surprise preservation, though its extent is not fully evident. This is where future studies need to be carried out to determine whether this effect of preservation applies to other audience affects. We are in the process of conducting further studies.

Overall, these findings may have fundamental consequences for the understanding of retelling and narratives, especially if similar results emerge for other affects tied to plot development. This is also related to our understanding of truthful retelling; some people may consider their retelling accurate and truthful, even if they changed facts, since they preserved the affective dimension of surprise. These results could provide additional insight to studies of the so-called flashbulb memory that yields especially vivid-seeming memories of surprising or unique events (Trankell, 1972; Winograd and Neisser, 2006; Welzer, 2010).

We are only beginning to understand the connection between affect and narrative. Storytelling and retelling is deeply intertwined in sharing affects, and in fact may share the common function of emotional communication. Whereas we often lack the vocabulary to capture fine-tuned degrees of affects, we are cognitively attentive to narrative affects with measured precision. Perhaps this affect sensitivity was a core evolutionary driver of the storytelling animal (Gottschall, 2012). If so, narratives and storytelling, including specific retelling activities might be considered a central feature of education in order to develop the affective side of our mental, cognitive, and social lives.

## Ethics Statement

This study was submitted to Indiana University Institutional Review Board (IRB) and was accepted as meeting the criteria of EXEMPT research as described in the United States Federal regulations at 45 CFR 46.101(b), paragraph(s) (2).

## Author Contributions

FB is the PI and was involved in all phases of the project. BL is a senior investigator who was involved in all phases except the original design of the studies. TL conducted the statistical analysis and was involved in various phases of project. ES-H was a co-designer of stimulus materials and involved in most phases of project. SW was a co-designer of stimulus materials and involved in first phase of data analysis.

## Conflict of Interest Statement

The authors declare that the research was conducted in the absence of any commercial or financial relationships that could be construed as a potential conflict of interest.
