# Supplementary material for: Fact vs. Affect in the Telephone Game: All Levels of Surprise Are Retold With High Accuracy, Even Independently of Facts
Source: Front Psychol. 2018 Nov 20;9:2210. doi: 10.3389/fpsyg.2018.02210 (PMC6255933; doi:10.3389/fpsyg.2018.02210)

**Appendix A. Stimulus Materials**

We used the following stories as stimulus materials. “***Insert***” marks the part that distinguishes the different variations.

1) Jason was a high school freshman. He was not very outgoing, did not do well in sports and was a big fan of science fiction TV programs. Near the beginning of the school year, he had an embarrassing moment playing football in gym class where the ball hit him in the head, causing his classmates to laugh at him and to tease him. By the middle of the semester, the school dance was coming up and all of Jason’s classmates were excited about it, but it did not interest Jason. Later, his best friend found a date, and began pressuring Jason to go as well, telling him that it would be fun. But Jason still remembered being laughed at by his whole class and did not want to get turned down. Jason did not ask anyone out. He stayed at home during the dance and avoided talking to his best friend about it. A few weeks later, a girl, who was new to town, transferred into Jason’s classes. Jason found her attractive and liked her more and more as time went on. But, he was too afraid to talk to her. One day, ***insert***. He mumbled that she looked nice and asked her if she would like to eat lunch with him.

- as he was doodling in class, she caught him drawing a perfect likeness of her (Jason A)

- as he was walking in front of her desk, he tripped on his shoelaces and fell right in front of her (Jason B)

- they were standing next to each other in a line (Jason C)

- as he was sitting in the classroom, he piled a bunch of different fruits on top of his head and danced over to the girl, while singing her name (Jason D)

- as he was walking down the hallway he saw the girl and noticed that they had on the same outfit (Jason E)

- he decided that to overcome his fear of talking to her he needed to assume an alternate identity. He dressed up as superhero and walked over to where she was sitting (Jason F)

- as he was walking behind her on the crosswalk to school, he noticed that a car was coming very fast towards them. He quickly ran and pushed her out of the way into safety (Jason G)

2) Sarah was a teenager living in a small town in the American Midwest. She was a good student who did well in all of her classes, except history. Her mother wanted her to do better in history and hired a student from the local college as a tutor. On a day with exceptionally good weather, Sarah decided to spend her afternoon playing softball with her friends instead of attending her tutoring session. The tutor called Sarah’s mother and told her about Sarah’s absence that afternoon. The news embarrassed Sarah’s mother, who scolded Sarah and lectured her on responsibility after she came home. Sarah did not think her skipping tutoring was a big deal since her mother did not have to pay for the missed lesson, she had good grades in all her other classes and she knew that her tutor was laidback and would not take her absence personally. Sarah and her mother had a fight and Sarah ran out of the house. She decided to go into the woods near her house. In the woods, ***insert***. That made her feel better and gave her the confidence to talk to her mother again. Her mother had been waiting at home and was busying herself preparing dinner when Sarah got back. Upon seeing her mother in the kitchen, Sarah apologized.

- she stomped around angrily and hit a tree (Sarah A)

- she read a book (Sarah B)

- she made a fire and burnt everything her mother had ever given her (Sarah C)

- she caught a strange looking snake (Sarah D)

- she dove into the pond and swam around with all her clothes on (Sarah E)

- she built a tree house and collected food for a month to stay there (Sarah F)

- she found an old racecar that worked and drove it at high speed into a tree (Sarah G)

3) Robert was a physics student attending a prestigious college on the East Coast. Even though he wanted to become a scientist, the college’s requirements demanded that he take at least one history class. Robert decided to get the class out of the way in his freshman year. He enrolled in the only one available, an American history class with a professor who had a reputation of being stern but knowledgeable. Robert felt that the class was interesting enough and did well in the assignments and on the midterm exam. Unfortunately for Robert, the history final exam was scheduled on the same day as the final for an important physics class. The day before the exams, Robert stayed up half of the night, mostly studying physics. After taking the physics test in the morning he felt that he had done well. At noon he sat down in class to take his history exam. He knew the exam would be difficult, but he was shocked to see how hard it was. He may not have studied enough, but this exam was simply not fair and he started sweating. With an hour left, he asked for a bathroom break and left the room. In the bathroom, ***insert***. Then he returned to the testing room to complete the exam.

- he pulled out his phone and searched the Internet for a couple exam questions (Robert A)

- he gave himself a pep talk while washing his hands and loudly sang his favorite song

regardless of the other people hearing him (Robert B)

-he splashed his face with water (Robert C)

- he did sprints in front of the stalls to get his brain going. While running, he hit his head on a door, but instead of confusing him, it seemed to cause everything to make sense (Robert D)

- a man he did not know gave him the textbook for his class with all relevant pages for the final marked (Robert E)

- he loudly asked the exam question to the mirror and a voice gave him the answer (Robert F)

- he found an envelope with his name on it. Inside was the answer key, signed “with love” from his teacher (Robert G).

**Appendix B. Correlations of Surprisingness of Stories (from Appendix A)**

The following are the median surprise ratings of each original variation and the averages of the surprise values of all third retellings for each original variation (based on median values of all reviewers for each specific retelling). The progression from A to G for each story shows the story specific gradation, but does not suggest a similarity between story sets (Jason B is not related or similar to Sarah B, etc.) Each individual story was evaluated in average by 7.8 (first iteration) to 10.2 participants (third iteration).

| Table A1  Median Surprise Ratings of Jason Stories | | | | | | | |
| --- | --- | --- | --- | --- | --- | --- | --- |
|  | A | B | C | D | E | F | G |
| Original | 2 | 2.3 | 3.2 | 4 | 4.6 | 4.95 | 5 |
| 1^st^ retelling | 2.09 | 2.29 | 2.37 | 4.31 | 2.85 | 4.49 | 4.49 |
| 2^nd^ retelling | 2.15 | 2.72 | 2.79 | 4.70 | 2.78 | 3.35 | 4.69 |
| 3rd retelling | 2.39 | 2.40 | 3.01 | 4.72 | 3.17 | 3.25 | 4.51 |
| *Note: surprisingness was rated on by participants with a slider and on a 7-point scale. The default slider position is 3.5.* | | | | | | | |

| Table A2  Pearson Correlation Matrix of Jason Retellings | | | | |
| --- | --- | --- | --- | --- |
|  | Original | 1^st^ Retelling | 2^nd^ Retelling | 3^rd^ Retelling |
| Original | 1 | - | - | - |
| 1^st^ retelling | .83 | 1 | - | - |
| 2^nd^ retelling | .65 | .87 | 1 | - |
| 3^rd^ retelling | .69 | .83 | .98 | 1 |
| *Note: there were a total of 97 3^rd^ retellings.* | | | | |

| Table A3  Median Surprise Ratings of Sarah Stories | | | | | | | |
| --- | --- | --- | --- | --- | --- | --- | --- |
|  | A | B | C | D | E | F | G |
| Original | 1 | 2 | 2.1 | 3.5 | 4.1 | 4.9 | 5 |
| 1^st^ retelling | 1.58 | 0.96 | 4.42 | 2.52 | 3.82 | 4.92 | 4.50 |
| 2^nd^ retelling | 1.52 | 1.23 | 4.34 | 2.14 | 3.67 | 5.05 | 4.57 |
| 3rd retelling | 1.98 | 1.65 | 3.25 | 2.43 | 3.66 | 4.85 | 4.61 |
| *Note: surprisingness was rated on by participants with a slider and on a 7-point scale. The default slider position is 3.5.* | | | | | | | |

| Table A4  Pearson Correlation Matrix of Sarah Retellings | | | | |
| --- | --- | --- | --- | --- |
|  | Original | 1^st^ Retelling | 2^nd^ Retelling | 3^rd^ Retelling |
| Original | 1 | - | - | - |
| 1^st^ retelling | .72 | 1 | - | - |
| 2^nd^ retelling | .72 | .99 | 1 | - |
| 3^rd^ retelling | .86 | .94 | .95 | 1 |
| *Note: there were a total of 94 3^rd^ retellings.* | | | | |

| Table A5  Median Surprise Ratings of Robert Stories | | | | | | | |
| --- | --- | --- | --- | --- | --- | --- | --- |
|  | A | B | C | D | E | F | G |
| Original | 1.6 | 2.5 | 4.3 | 5.1 | 5.25 | 6 | 6.1 |
| 1^st^ retelling | 0.55 | 2.10 | 2.72 | 4.36 | 4.24 | 4.62 | 5.48 |
| 2^nd^ retelling | 1.12 | 1.99 | 1.92 | 4.03 | 4.25 | 4.45 | 4.95 |
| 3rd retelling | 1.05 | 2.26 | 3.42 | 3.94 | 3.21 | 4.13 | 5.49 |
| *Note: surprisingness was rated on by participants with a slider and on a 7-point scale. The default slider position is 3.5.* | | | | | | | |

| Table A6  Pearson Correlation Matrix of Robert Retellings | | | | |
| --- | --- | --- | --- | --- |
|  | Original | 1^st^ Retelling | 2^nd^ Retelling | 3^rd^ Retelling |
| Original | 1 | - | - | - |
| 1^st^ retelling | .95 | 1 | - | - |
| 2^nd^ retelling | .95 | .97 | 1 | - |
| 3^rd^ retelling | .93 | .94 | .86 | 1 |
| *Note: there were a total of 94 3^rd^ retellings.* | | | | |

|  | | | | | | | | | |
| --- | --- | --- | --- | --- | --- | --- | --- | --- | --- |
| Table A7  Pearson Correlations of Median Surprisingness between Retelling Iterations | | | | | | | | | |
|  | Jason  *r* | Jason *n* | Jason *p* | Sarah  *r* | Sarah *n* | Sarah  *p* | Robert *r* | Robert *n* | Robert  *p* |
| Original to 1^st^ retelling | .85 | 97 | .0144* | .73 | 94 | .0625 | .95 | 94 | .0010** |
| 1^st^ to 2^nd^ retelling | .87 | 97 | .0110* | .95 | 94 | .0009*** | .98 | 94 | .0001*** |
| 2^nd^ to 3^rd^ retelling | .89 | 97 | .0070** | .84 | 94 | .0185* | .87 | 94 | .0105* |
| *Note: *P < .05 or better, ** P < .01 or better, ***P < .001 or better.* | | | | | | | | | |

| Table A8  Pearson Correlations of All Individual Stories between Iterations | | | | | | | | | |
| --- | --- | --- | --- | --- | --- | --- | --- | --- | --- |
|  | Jason  *r* | Jason *n* | Jason *p* | Sarah  *r* | Sarah *n* | Sarah  *p* | Robert *r* | Robert *n* | Robert  *p* |
| Original to 1^st^ retelling | .60 | 97 | 6.731e-13*** | .54 | 94 | 2.83e-10*** | .69 | 94 | 2.2e-16*** |
| 1^st^ to 2^nd^ retelling | .53 | 97 | 4.733e-8*** | .78 | 94 | 2.2e-16*** | .74 | 94 | 2.2e-16*** |
| 2^nd^ to 3^rd^ retelling | .60 | 97 | 2.084e-10** | .77 | 94 | 2.2e-16*** | .82 | 94 | 2.2e-16*** |
| *Note: ***P < .001 or better.* | | | | | | | | | |

**Appendix C.**

We used the shortened stimulus stories (Appendix D) and asked 264 participants (including 42 raters also counted in Appendix D as raters for “surprise” of the original short stories) to rate 15 randomly selected stories of the 21 stories from according to one of the following criteria: surprise, probability, eventfulness, gap in cause and effect, event strength, interest/suspense, emotional excitement, or vicariousness. These criteria were used in accordance with standard literature from either psychological studies on surprise (probability, gap in cause and effect, emotional excitement; Reisenzein, 2000; Maguire & Keane, 2011; Teigen & Keren, 2003) or narratological literature (eventfulness, event strength, interest/suspense, or vicariousness; Schmid, 2010; Keen, 2006). Each criterion was rated by 30-42 participants. We did not define the terms, but asked simple questions (“How probable is the story?”, “How emotionally exciting is the story?” or “How strong is the event?”). Only in the case of “vicariousness” did we word the question differently (“How intensely do you feel like the character?”). Surprise, eventfulness and probability were the most strongly correlated; in fact, surprise and probability were hardly distinguished at all (-.97). Participants may have been unclear about the meaning of “eventfulness” and “event strength” – two concepts sometimes used in narratology – which we measured without explanations.)

| Table A9  Pearson Correlations of Surprise to Different Measures | | | | | | | | | |
| --- | --- | --- | --- | --- | --- | --- | --- | --- | --- |
|  | Surprise | Probability | Eventfulness | Gap in cause  & effect | Event strength | Suspense/  interest | Emotional excitement | Vicariousness |  |
| Surprise | 1 |  |  |  |  |  |  |  |  |
| Probability | -.97 | 1 |  |  |  |  |  |  |  |
| Eventfulness | .84 | .79 | 1 |  |  |  |  |  |  |
| Gap in cause & effect | .69 | .62 | .47 | 1 |  |  |  |  |  |
| Event strength | .6 | .51 | .75 | .47 | 1 |  |  |  |  |
| Interesting/  Suspenseful | .58 | .59 | .51 | .17 | .43 | 1 |  |  |  |
| Emotional excitement | -.1 | -.17 | 0 | 0 | .26 | -.18 | 1 |  |  |
| Vicariousness | -.76 | -.76 | -.54 | -.54 | -.22 | -.28 | .18 | 1 |  |

**Appendix D. Surprisingness Correlations of Short Stories**

We shortened the stimulus materials from Appendix A in the following way. “***Insert***” marks the part that varies between the variations. The variations are identical to those in Appendix A.

1) Jason liked a girl in his class. He was very shy, however, and was too afraid to talk to her. One day, ***insert***. He mumbled that she looked nice and asked her if she would like to eat lunch with him.

2) Sarah had a fight with her mother. She ran out of the house. She decided to go into the woods. In the woods, ***insert***. That made her feel better and gave her the confidence to talk to her mother again. After that, she went back home and apologized.

3) Robert sat down in class to take his final exam. He knew the exam would be difficult, but he was shocked to see how hard it was. He may not have studied enough, but this exam was simply not fair and he started sweating. With an hour left, he asked for a bathroom break and left the room. In the bathroom, ***insert***. Then he returned to the testing room to complete the exam.

Story names from the short texts in Appendix D are consistent with the names in Appendices A and B. Again, we used median ratings for each story and calculated averages of all retellings derived from the same original variation.

| Table A10  Median original and Median 3rd-Retelling Surprise for Jason Stories | | | | | | | |
| --- | --- | --- | --- | --- | --- | --- | --- |
|  | Jason C | Jason A | Jason B | Jason E | Jason G | Jason F | Jason D |
| Original | 1.05 | 2.5 | 2.7 | 3.05 | 5 | 5.5 | 6 |
| 3rd retelling | 1.54 | 2.1 | 3.03 | 2.32 | 3.45 | 3.49 | 4.53 |
| *Note: there were 127 3^rd^ retellings. Surprisingness was rated on by participants with a slider and on a 7-point scale. The default slider position is 3.5. The Pearson correlation between original and 3^rd^ was .93. Spearman’s rho was .96.* | | | | | | | |

| Table A11  Median original and Median 3rd-Retelling Surprise for Sarah Stories | | | | | | | |
| --- | --- | --- | --- | --- | --- | --- | --- |
|  | Sarah B | Sarah A | Sarah E | Sarah D | Sarah F | Sarah C | Sarah G |
| Original | 1.05 | 1.85 | 4.05 | 4.4 | 5.15 | 5.25 | 6.25 |
| 3rd retelling | 2.3 | 2.1 | 3.35 | 4.32 | 4.78 | 4.24 | 5.66 |
| *Note: there were 117 3^rd^ retellings. Surprisingness was rated on by participants with a slider and on a 7-point scale. The default slider position is 3.5. The Pearson correlation between original and 3^rd^ was .95. Spearman’s rho was .86.* | | | | | | | |

| Table A12  Median original and Median 3rd-Retelling Surprise for Robert Stories | | | | | | | |
| --- | --- | --- | --- | --- | --- | --- | --- |
|  | Robert C | Robert A | Robert B | Robert E | Robert D | Robert  F | Robert G |
| Original | 0.45 | 1.45 | 3.05 | 5.1 | 5.15 | 6.15 | 6.3 |
| 3rd retelling | 0.95 | 2.77 | 2.1 | 4.84 | 4.91 | 5.18 | 5.73 |
| *Note: there were 133 3^rd^ retellings. Surprisingness was rated on by participants with a slider and on a 7-point scale. The default slider position is 3.5. The Pearson correlation between original and 3^rd^ was .95. Spearman’s rho was .96.* | | | | | | | |

**Appendix E. Estimate of Surprisingness in the Bayesian ANOVA by Story**

In this appendix we present the posterior estimates of the surprise ratings for each story variation. In each of the three plots below, we show the posterior predicted mean surprisingness ratings for the Jason, Sarah, and Robert stories respectively, on the original 7-point surprisingness scale. Note that these surprisingness rating estimates are across all iterations. The vertical bars represent the 95% HDI for each story, and the point at the center is the modal estimate for each story. These plots indicate that our model has high precision on the estimates of the mean surprisingness ratings, and that the model appears to capture the tendencies observed in Fig. 2.


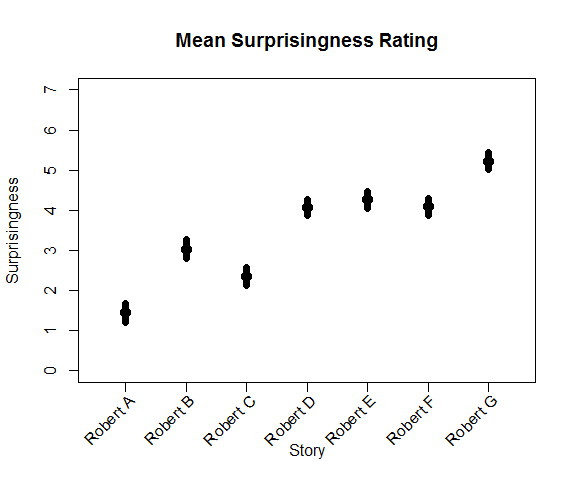


**
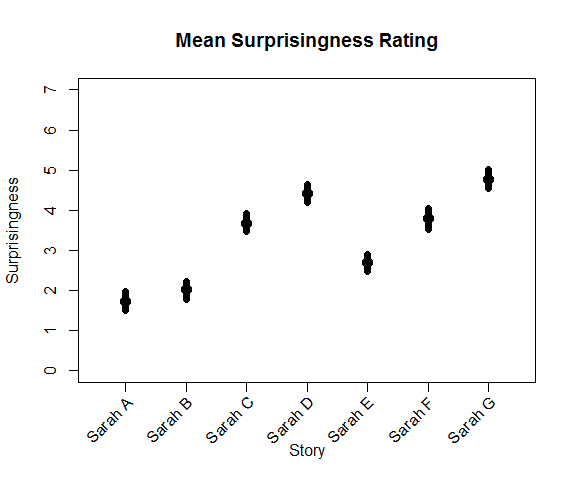
**


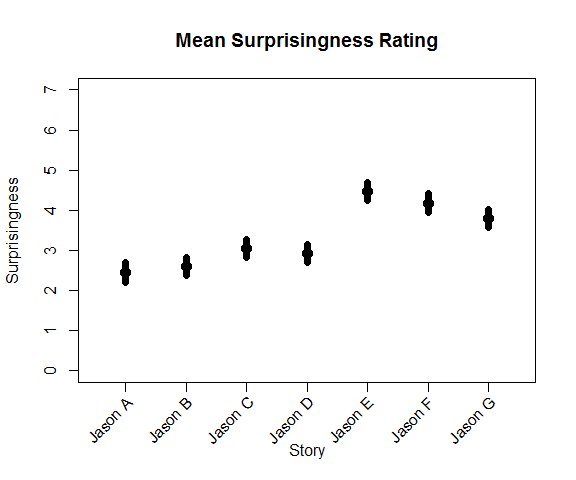

Supplement: Supplementary file 1 [file Data_Sheet_1.docx]
